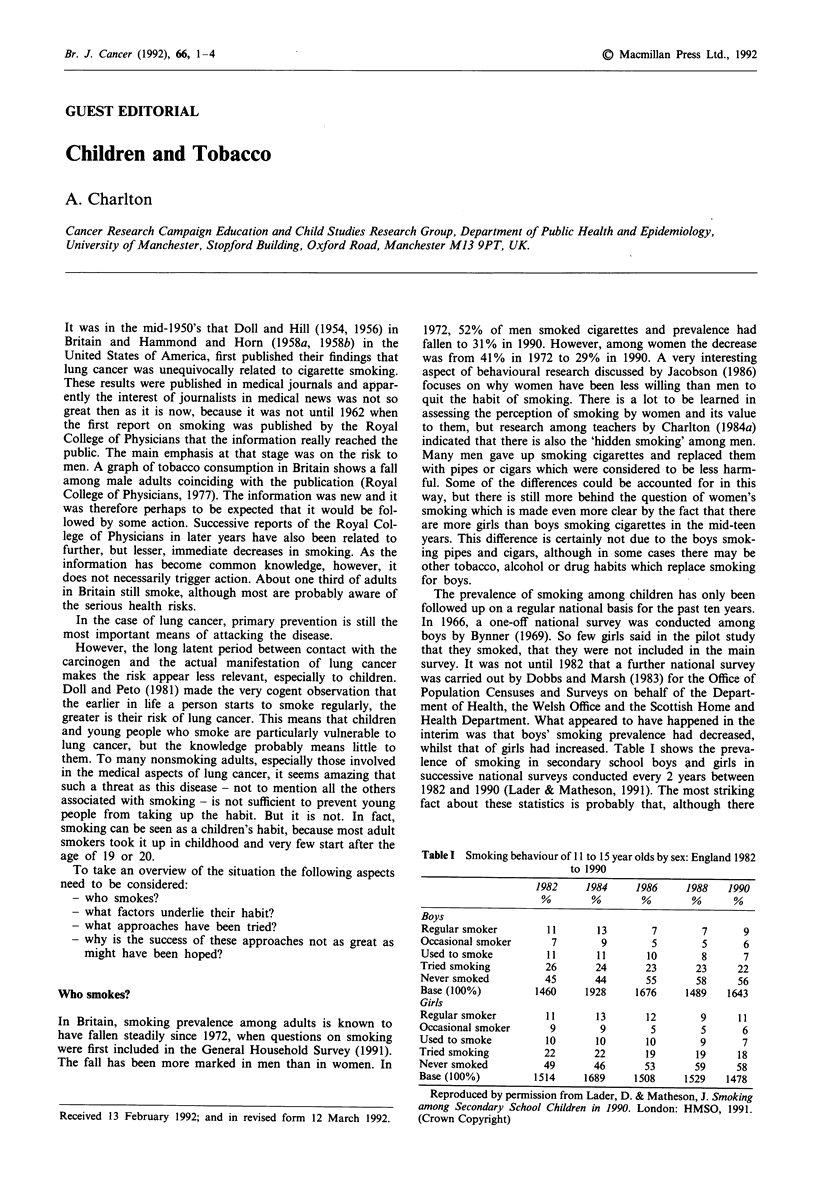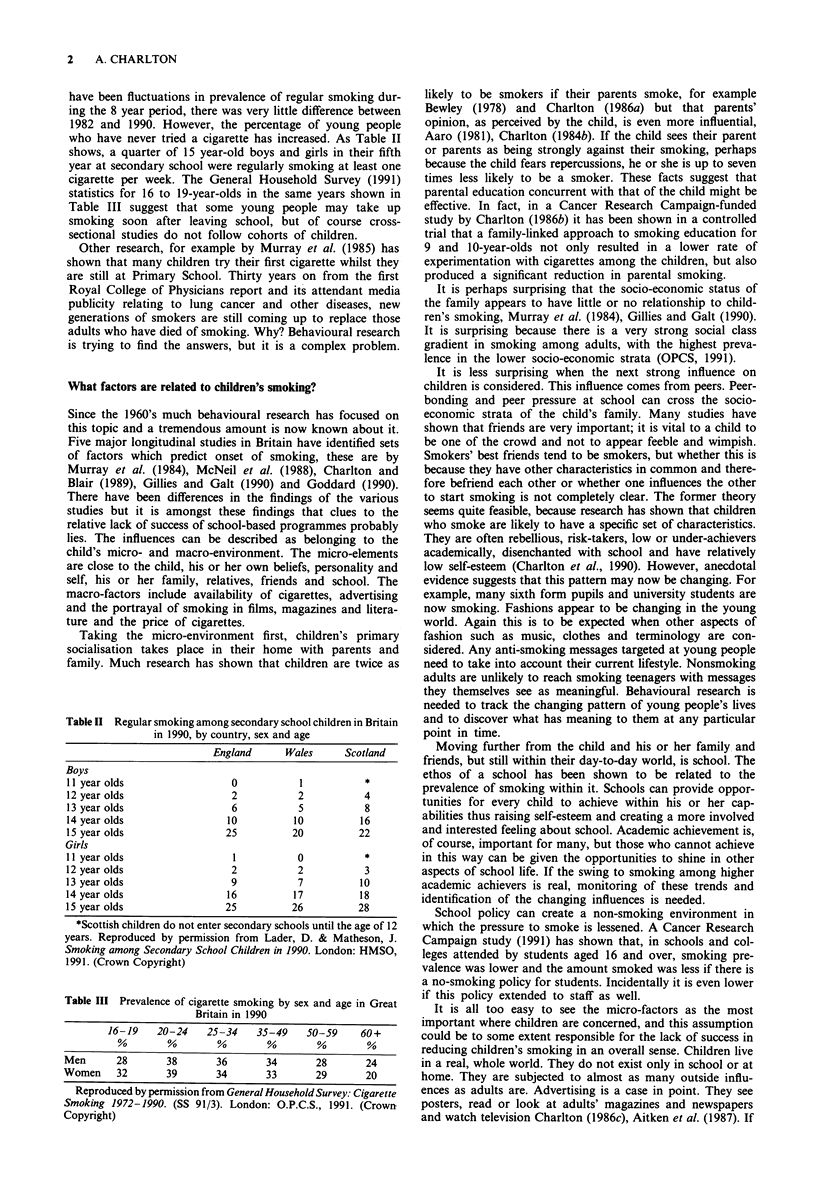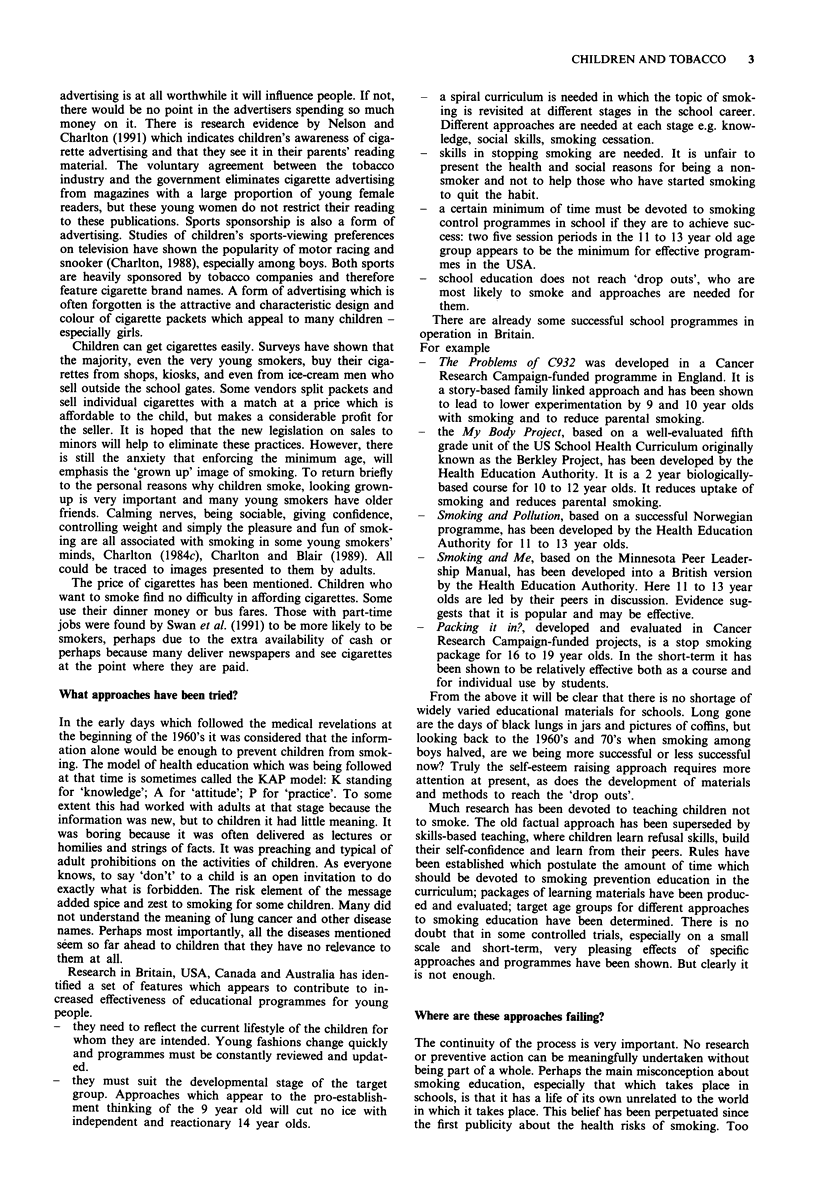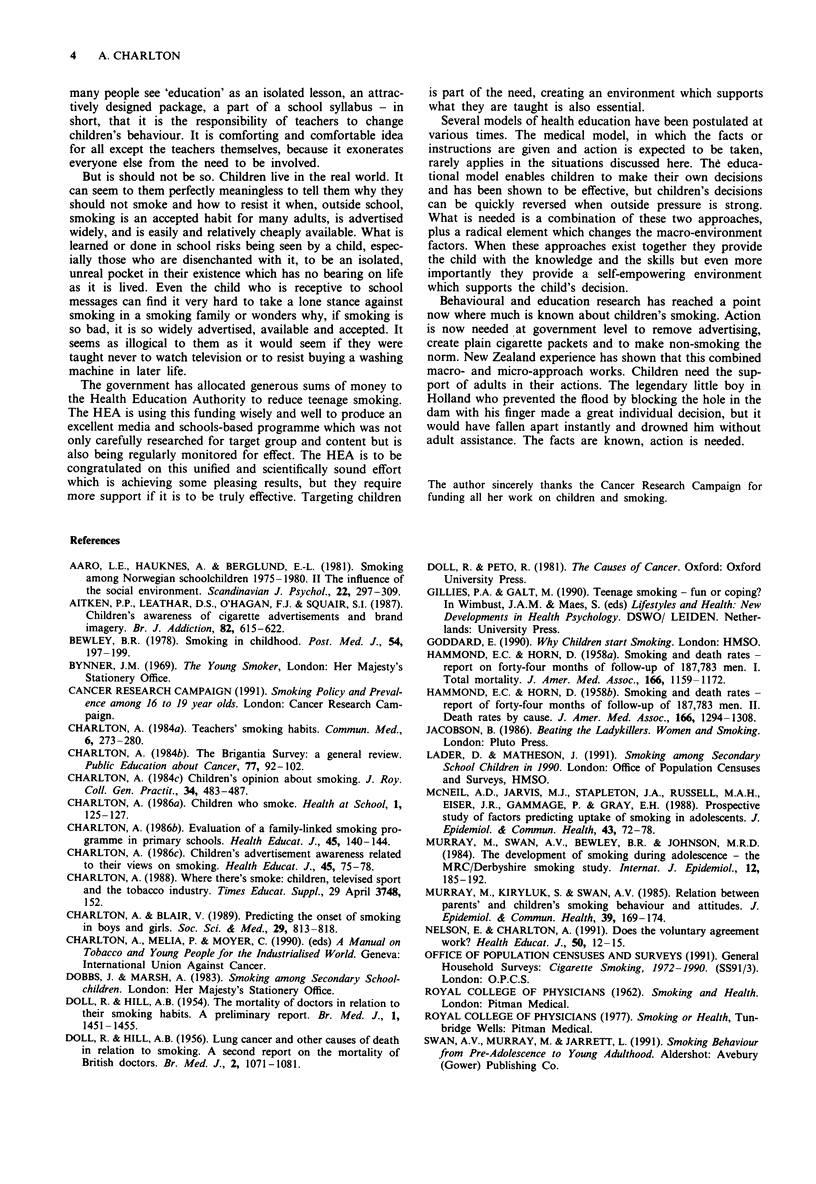# Children and tobacco.

**DOI:** 10.1038/bjc.1992.207

**Published:** 1992-07

**Authors:** A. Charlton


Br. J. Cancer (1992), 66, 1-4 © Macmillan Press Ltd., 1992

GUEST EDITORIAL

### Children and Tobacco

A. Charlton

*Cancer Research Campaign Education and Child Studies Research Group, Department of Public Health and Epidemiology, University of Manchester, Stopford Building, Oxford Road, Manchester M13 9PT, UK.*

It was in the mid-1950's that Doll and Hill (1954, 1956) in Britain and Hammond and Horn (1958*a*, 1958*b*) in the United States of America, first published their findings that lung cancer was unequivocally related to cigarette smoking. These results were published in medical journals and apparently the interest of journalists in medical news was not so great then as it is now, because it was not until 1962 when the first report on smoking was published by the Royal College of Physicians that the information really reached the public. The main emphasis at that stage was on the risk to men. A graph of tobacco consumption in Britain shows a fall among male adults coinciding with the publication (Royal College of Physicians, 1977). The information was new and it was therefore perhaps to be expected that it would be followed by some action. Successive reports of the Royal College of Physicians in later years have also been related to further, but lesser, immediate decreases in smoking. As the information has become common knowledge, however, it does not necessarily trigger action. About one third of adults in Britain still smoke, although most are probably aware of the serious health risks.

In the case of lung cancer, primary prevention is still the most important means of attacking the disease.

However, the long latent period between contact with the carcinogen and the actual manifestation of lung cancer makes the risk appear less relevant, especially to children. Doll and Peto (1981) made the very cogent observation that the earlier in life a person starts to smoke regularly, the greater is their risk of lung cancer. This means that children and young people who smoke are particularly vulnerable to lung cancer, but the knowledge probably means little to them. To many nonsmoking adults, especially those involved in the medical aspects of lung cancer, it seems amazing that such a threat as this disease – not to mention all the others associated with smoking – is not sufficient to prevent young people from taking up the habit. But it is not. In fact, smoking can be seen as a children's habit, because most adult smokers took it up in childhood and very few start after the age of 19 or 20.

To take an overview of the situation the following aspects need to be considered:

- who smokes?
- what factors underlie their habit?
- what approaches have been tried?
- why is the success of these approaches not as great as might have been hoped?

### Who smokes?

In Britain, smoking prevalence among adults is known to have fallen steadily since 1972, when questions on smoking were first included in the General Household Survey (1991). The fall has been more marked in men than in women. In 1972, 52% of men smoked cigarettes and prevalence had fallen to 31% in 1990. However, among women the decrease was from 41% in 1972 to 29% in 1990. A very interesting aspect of behavioural research discussed by Jacobson (1986) focuses on why women have been less willing than men to quit the habit of smoking. There is a lot to be learned in assessing the perception of smoking by women and its value to them, but research among teachers by Charlton (1984*a*) indicated that there is also the 'hidden smoking' among men. Many men gave up smoking cigarettes and replaced them with pipes or cigars which were considered to be less harmful. Some of the differences could be accounted for in this way, but there is still more behind the question of women's smoking which is made even more clear by the fact that there are more girls than boys smoking cigarettes in the mid-teen years. This difference is certainly not due to the boys smoking pipes and cigars, although in some cases there may be other tobacco, alcohol or drug habits which replace smoking for boys.

The prevalence of smoking among children has only been followed up on a regular national basis for the past ten years. In 1966, a one-off national survey was conducted among boys by Bynner (1969). So few girls said in the pilot study that they smoked, that they were not included in the main survey. It was not until 1982 that a further national survey was carried out by Dobbs and Marsh (1983) for the Office of Population Censuses and Surveys on behalf of the Department of Health, the Welsh Office and the Scottish Home and Health Department. What appeared to have happened in the interim was that boys' smoking prevalence had decreased, whilst that of girls had increased. Table I shows the prevalence of smoking in secondary school boys and girls in successive national surveys conducted every 2 years between 1982 and 1990 (Lader & Matheson, 1991). The most striking fact about these statistics is probably that, although there

**Table I** Smoking behaviour of 11 to 15 year olds by sex: England 1982 to 1990

| | *1982* % | *1984* % | *1986* % | *1988* % | *1990* % |
|---|---|---|---|---|---|
| *Boys* | | | | | |
| Regular smoker | 11 | 13 | 7 | 7 | 9 |
| Occasional smoker | 7 | 9 | 5 | 5 | 6 |
| Used to smoke | 11 | 11 | 10 | 8 | 7 |
| Tried smoking | 26 | 24 | 23 | 23 | 22 |
| Never smoked | 45 | 44 | 55 | 58 | 56 |
| Base (100%) | 1460 | 1928 | 1676 | 1489 | 1643 |
| *Girls* | | | | | |
| Regular smoker | 11 | 13 | 12 | 9 | 11 |
| Occasional smoker | 9 | 9 | 5 | 5 | 6 |
| Used to smoke | 10 | 10 | 10 | 9 | 7 |
| Tried smoking | 22 | 22 | 19 | 19 | 18 |
| Never smoked | 49 | 46 | 53 | 59 | 58 |
| Base (100%) | 1514 | 1689 | 1508 | 1529 | 1478 |

Reproduced by permission from Lader, D. & Matheson, J. *Smoking among Secondary School Children in 1990*. London: HMSO, 1991. (Crown Copyright)

Received 13 February 1992; and in revised form 12 March 1992.

have been fluctuations in prevalence of regular smoking during the 8 year period, there was very little difference between 1982 and 1990. However, the percentage of young people who have never tried a cigarette has increased. As Table II shows, a quarter of 15 year-old boys and girls in their fifth year at secondary school were regularly smoking at least one cigarette per week. The General Household Survey (1991) statistics for 16 to 19-year-olds in the same years shown in Table III suggest that some young people may take up smoking soon after leaving school, but of course cross-sectional studies do not follow cohorts of children.

Other research, for example by Murray *et al.* (1985) has shown that many children try their first cigarette whilst they are still at Primary School. Thirty years on from the first Royal College of Physicians report and its attendant media publicity relating to lung cancer and other diseases, new generations of smokers are still coming up to replace those adults who have died of smoking. Why? Behavioural research is trying to find the answers, but it is a complex problem.

### What factors are related to children's smoking?

Since the 1960's much behavioural research has focused on this topic and a tremendous amount is now known about it. Five major longitudinal studies in Britain have identified sets of factors which predict onset of smoking, these are by Murray *et al.* (1984), McNeil *et al.* (1988), Charlton and Blair (1989), Gillies and Galt (1990) and Goddard (1990). There have been differences in the findings of the various studies but it is amongst these findings that clues to the relative lack of success of school-based programmes probably lies. The influences can be described as belonging to the child's micro- and macro-environment. The micro-elements are close to the child, his or her own beliefs, personality and self, his or her family, relatives, friends and school. The macro-factors include availability of cigarettes, advertising and the portrayal of smoking in films, magazines and literature and the price of cigarettes.

Taking the micro-environment first, children's primary socialisation takes place in their home with parents and family. Much research has shown that children are twice as likely to be smokers if their parents smoke, for example Bewley (1978) and Charlton (1986*a*) but that parents' opinion, as perceived by the child, is even more influential, Aaro (1981), Charlton (1984*b*). If the child sees their parent or parents as being strongly against their smoking, perhaps because the child fears repercussions, he or she is up to seven times less likely to be a smoker. These facts suggest that parental education concurrent with that of the child might be effective. In fact, in a Cancer Research Campaign-funded study by Charlton (1986*b*) it has been shown in a controlled trial that a family-linked approach to smoking education for 9 and 10-year-olds not only resulted in a lower rate of experimentation with cigarettes among the children, but also produced a significant reduction in parental smoking.

It is perhaps surprising that the socio-economic status of the family appears to have little or no relationship to children's smoking, Murray *et al.* (1984), Gillies and Galt (1990). It is surprising because there is a very strong social class gradient in smoking among adults, with the highest prevalence in the lower socio-economic strata (OPCS, 1991).

It is less surprising when the next strong influence on children is considered. This influence comes from peers. Peer-bonding and peer pressure at school can cross the socio-economic strata of the child's family. Many studies have shown that friends are very important; it is vital to a child to be one of the crowd and not to appear feeble and wimpish. Smokers' best friends tend to be smokers, but whether this is because they have other characteristics in common and therefore befriend each other or whether one influences the other to start smoking is not completely clear. The former theory seems quite feasible, because research has shown that children who smoke are likely to have a specific set of characteristics. They are often rebellious, risk-takers, low or under-achievers academically, disenchanted with school and have relatively low self-esteem (Charlton *et al.*, 1990). However, anecdotal evidence suggests that this pattern may now be changing. For example, many sixth form pupils and university students are now smoking. Fashions appear to be changing in the young world. Again this is to be expected when other aspects of fashion such as music, clothes and terminology are considered. Any anti-smoking messages targeted at young people need to take into account their current lifestyle. Nonsmoking adults are unlikely to reach smoking teenagers with messages they themselves see as meaningful. Behavioural research is needed to track the changing pattern of young people's lives and to discover what has meaning to them at any particular point in time.

Moving further from the child and his or her family and friends, but still within their day-to-day world, is school. The ethos of a school has been shown to be related to the prevalence of smoking within it. Schools can provide opportunities for every child to achieve within his or her capabilities thus raising self-esteem and creating a more involved and interested feeling about school. Academic achievement is, of course, important for many, but those who cannot achieve in this way can be given the opportunities to shine in other aspects of school life. If the swing to smoking among higher academic achievers is real, monitoring of these trends and identification of the changing influences is needed.

School policy can create a non-smoking environment in which the pressure to smoke is lessened. A Cancer Research Campaign study (1991) has shown that, in schools and colleges attended by students aged 16 and over, smoking prevalence was lower and the amount smoked was less if there is a no-smoking policy for students. Incidentally it is even lower if this policy extended to staff as well.

It is all too easy to see the micro-factors as the most important where children are concerned, and this assumption could be to some extent responsible for the lack of success in reducing children's smoking in an overall sense. Children live in a real, whole world. They do not exist only in school or at home. They are subjected to almost as many outside influences as adults are. Advertising is a case in point. They see posters, read or look at adults' magazines and newspapers and watch television Charlton (1986*c*), Aitken *et al.* (1987). If

**Table II** Regular smoking among secondary school children in Britain in 1990, by country, sex and age

| | *England* | *Wales* | *Scotland* |
|---|---|---|---|
| *Boys* | | | |
| 11 year olds | 0 | 1 | * |
| 12 year olds | 2 | 2 | 4 |
| 13 year olds | 6 | 5 | 8 |
| 14 year olds | 10 | 10 | 16 |
| 15 year olds | 25 | 20 | 22 |
| *Girls* | | | |
| 11 year olds | 1 | 0 | * |
| 12 year olds | 2 | 2 | 3 |
| 13 year olds | 9 | 7 | 10 |
| 14 year olds | 16 | 17 | 18 |
| 15 year olds | 25 | 26 | 28 |

*Scottish children do not enter secondary schools until the age of 12 years. Reproduced by permission from Lader, D. & Matheson, J. *Smoking among Secondary School Children in 1990*. London: HMSO, 1991. (Crown Copyright)

**Table III** Prevalence of cigarette smoking by sex and age in Great Britain in 1990

| | *16–19* % | *20–24* % | *25–34* % | *35–49* % | *50–59* % | *60+* % |
|---|---|---|---|---|---|---|
| Men | 28 | 38 | 36 | 34 | 28 | 24 |
| Women | 32 | 39 | 34 | 33 | 29 | 20 |

Reproduced by permission from *General Household Survey: Cigarette Smoking 1972–1990*. (SS 91/3). London: O.P.C.S., 1991. (Crown Copyright)

advertising is at all worthwhile it will influence people. If not, there would be no point in the advertisers spending so much money on it. There is research evidence by Nelson and Charlton (1991) which indicates children's awareness of cigarette advertising and that they see it in their parents' reading material. The voluntary agreement between the tobacco industry and the government eliminates cigarette advertising from magazines with a large proportion of young female readers, but these young women do not restrict their reading to these publications. Sports sponsorship is also a form of advertising. Studies of children's sports-viewing preferences on television have shown the popularity of motor racing and snooker (Charlton, 1988), especially among boys. Both sports are heavily sponsored by tobacco companies and therefore feature cigarette brand names. A form of advertising which is often forgotten is the attractive and characteristic design and colour of cigarette packets which appeal to many children – especially girls.

Children can get cigarettes easily. Surveys have shown that the majority, even the very young smokers, buy their cigarettes from shops, kiosks, and even from ice-cream men who sell outside the school gates. Some vendors split packets and sell individual cigarettes with a match at a price which is affordable to the child, but makes a considerable profit for the seller. It is hoped that the new legislation on sales to minors will help to eliminate these practices. However, there is still the anxiety that enforcing the minimum age, will emphasis the 'grown up' image of smoking. To return briefly to the personal reasons why children smoke, looking grown-up is very important and many young smokers have older friends. Calming nerves, being sociable, giving confidence, controlling weight and simply the pleasure and fun of smoking are all associated with smoking in some young smokers' minds, Charlton (1984*c*), Charlton and Blair (1989). All could be traced to images presented to them by adults.

The price of cigarettes has been mentioned. Children who want to smoke find no difficulty in affording cigarettes. Some use their dinner money or bus fares. Those with part-time jobs were found by Swan *et al.* (1991) to be more likely to be smokers, perhaps due to the extra availability of cash or perhaps because many deliver newspapers and see cigarettes at the point where they are paid.

**What approaches have been tried?**

In the early days which followed the medical revelations at the beginning of the 1960's it was considered that the information alone would be enough to prevent children from smoking. The model of health education which was being followed at that time is sometimes called the KAP model: K standing for 'knowledge'; A for 'attitude'; P for 'practice'. To some extent this had worked with adults at that stage because the information was new, but to children it had little meaning. It was boring because it was often delivered as lectures or homilies and strings of facts. It was preaching and typical of adult prohibitions on the activities of children. As everyone knows, to say 'don't' to a child is an open invitation to do exactly what is forbidden. The risk element of the message added spice and zest to smoking for some children. Many did not understand the meaning of lung cancer and other disease names. Perhaps most importantly, all the diseases mentioned seem so far ahead to children that they have no relevance to them at all.

Research in Britain, USA, Canada and Australia has identified a set of features which appears to contribute to increased effectiveness of educational programmes for young people.

- they need to reflect the current lifestyle of the children for whom they are intended. Young fashions change quickly and programmes must be constantly reviewed and updated.
- they must suit the developmental stage of the target group. Approaches which appear to the pro-establishment thinking of the 9 year old will cut no ice with independent and reactionary 14 year olds.
- a spiral curriculum is needed in which the topic of smoking is revisited at different stages in the school career. Different approaches are needed at each stage e.g. knowledge, social skills, smoking cessation.
- skills in stopping smoking are needed. It is unfair to present the health and social reasons for being a non-smoker and not to help those who have started smoking to quit the habit.
- a certain minimum of time must be devoted to smoking control programmes in school if they are to achieve success: two five session periods in the 11 to 13 year old age group appears to be the minimum for effective programmes in the USA.
- school education does not reach 'drop outs', who are most likely to smoke and approaches are needed for them.

There are already some successful school programmes in operation in Britain.

For example

- *The Problems of C932* was developed in a Cancer Research Campaign-funded programme in England. It is a story-based family linked approach and has been shown to lead to lower experimentation by 9 and 10 year olds with smoking and to reduce parental smoking.
- the *My Body Project*, based on a well-evaluated fifth grade unit of the US School Health Curriculum originally known as the Berkley Project, has been developed by the Health Education Authority. It is a 2 year biologically-based course for 10 to 12 year olds. It reduces uptake of smoking and reduces parental smoking.
- *Smoking and Pollution*, based on a successful Norwegian programme, has been developed by the Health Education Authority for 11 to 13 year olds.
- *Smoking and Me*, based on the Minnesota Peer Leadership Manual, has been developed into a British version by the Health Education Authority. Here 11 to 13 year olds are led by their peers in discussion. Evidence suggests that it is popular and may be effective.
- *Packing it in?*, developed and evaluated in Cancer Research Campaign-funded projects, is a stop smoking package for 16 to 19 year olds. In the short-term it has been shown to be relatively effective both as a course and for individual use by students.

From the above it will be clear that there is no shortage of widely varied educational materials for schools. Long gone are the days of black lungs in jars and pictures of coffins, but looking back to the 1960's and 70's when smoking among boys halved, are we being more successful or less successful now? Truly the self-esteem raising approach requires more attention at present, as does the development of materials and methods to reach the 'drop outs'.

Much research has been devoted to teaching children not to smoke. The old factual approach has been superseded by skills-based teaching, where children learn refusal skills, build their self-confidence and learn from their peers. Rules have been established which postulate the amount of time which should be devoted to smoking prevention education in the curriculum; packages of learning materials have been produced and evaluated; target age groups for different approaches to smoking education have been determined. There is no doubt that in some controlled trials, especially on a small scale and short-term, very pleasing effects of specific approaches and programmes have been shown. But clearly it is not enough.

**Where are these approaches failing?**

The continuity of the process is very important. No research or preventive action can be meaningfully undertaken without being part of a whole. Perhaps the main misconception about smoking education, especially that which takes place in schools, is that it has a life of its own unrelated to the world in which it takes place. This belief has been perpetuated since the first publicity about the health risks of smoking. Too many people see 'education' as an isolated lesson, an attractively designed package, a part of a school syllabus – in short, that it is the responsibility of teachers to change children's behaviour. It is comforting and comfortable idea for all except the teachers themselves, because it exonerates everyone else from the need to be involved.

But is should not be so. Children live in the real world. It can seem to them perfectly meaningless to tell them why they should not smoke and how to resist it when, outside school, smoking is an accepted habit for many adults, is advertised widely, and is easily and relatively cheaply available. What is learned or done in school risks being seen by a child, especially those who are disenchanted with it, to be an isolated, unreal pocket in their existence which has no bearing on life as it is lived. Even the child who is receptive to school messages can find it very hard to take a lone stance against smoking in a smoking family or wonders why, if smoking is so bad, it is so widely advertised, available and accepted. It seems as illogical to them as it would seem if they were taught never to watch television or to resist buying a washing machine in later life.

The government has allocated generous sums of money to the Health Education Authority to reduce teenage smoking. The HEA is using this funding wisely and well to produce an excellent media and schools-based programme which was not only carefully researched for target group and content but is also being regularly monitored for effect. The HEA is to be congratulated on this unified and scientifically sound effort which is achieving some pleasing results, but they require more support if it is to be truly effective. Targeting children is part of the need, creating an environment which supports what they are taught is also essential.

Several models of health education have been postulated at various times. The medical model, in which the facts or instructions are given and action is expected to be taken, rarely applies in the situations discussed here. The educational model enables children to make their own decisions and has been shown to be effective, but children's decisions can be quickly reversed when outside pressure is strong. What is needed is a combination of these two approaches, plus a radical element which changes the macro-environment factors. When these approaches exist together they provide the child with the knowledge and the skills but even more importantly they provide a self-empowering environment which supports the child's decision.

Behavioural and education research has reached a point now where much is known about children's smoking. Action is now needed at government level to remove advertising, create plain cigarette packets and to make non-smoking the norm. New Zealand experience has shown that this combined macro- and micro-approach works. Children need the support of adults in their actions. The legendary little boy in Holland who prevented the flood by blocking the hole in the dam with his finger made a great individual decision, but it would have fallen apart instantly and drowned him without adult assistance. The facts are known, action is needed.

The author sincerely thanks the Cancer Research Campaign for funding all her work on children and smoking.

**References**

AARO, L.E., HAUKNES, A. & BERGLUND, E.-L. (1981). Smoking among Norwegian schoolchildren 1975–1980. II The influence of the social environment. *Scandinavian J. Psychol.*, **22**, 297–309.

AITKEN, P.P., LEATHAR, D.S., O'HAGAN, F.J. & SQUAIR, S.I. (1987). Children's awareness of cigarette advertisements and brand imagery. *Br. J. Addiction*, **82**, 615–622.

BEWLEY, B.R. (1978). Smoking in childhood. *Post. Med. J.*, **54**, 197–199.

BYNNER, J.M. (1969). *The Young Smoker*, London: Her Majesty's Stationery Office.

CANCER RESEARCH CAMPAIGN (1991). *Smoking Policy and Prevalence among 16 to 19 year olds*. London: Cancer Research Campaign.

CHARLTON, A. (1984*a*). Teachers' smoking habits. *Commun. Med.*, **6**, 273–280.

CHARLTON, A. (1984*b*). The Brigantia Survey: a general review. *Public Education about Cancer*, **77**, 92–102.

CHARLTON, A. (1984*c*) Children's opinion about smoking. *J. Roy. Coll. Gen. Practit.*, **34**, 483–487.

CHARLTON, A. (1986*a*). Children who smoke. *Health at School*, **1**, 125–127.

CHARLTON, A. (1986*b*). Evaluation of a family-linked smoking programme in primary schools. *Health Educat. J.*, **45**, 140–144.

CHARLTON, A. (1986*c*). Children's advertisement awareness related to their views on smoking. *Health Educat. J.*, **45**, 75–78.

CHARLTON, A. (1988). Where there's smoke: children, televised sport and the tobacco industry. *Times Educat. Suppl.*, 29 April **3748**, 152.

CHARLTON, A. & BLAIR, V. (1989). Predicting the onset of smoking in boys and girls. *Soc. Sci. & Med.*, **29**, 813–818.

CHARLTON, A., MELIA, P. & MOYER, C. (1990). (eds) *A Manual on Tobacco and Young People for the Industrialised World*. Geneva: International Union Against Cancer.

DOBBS, J. & MARSH, A. (1983). *Smoking among Secondary Schoolchildren*. London: Her Majesty's Stationery Office.

DOLL, R. & HILL, A.B. (1954). The mortality of doctors in relation to their smoking habits. A preliminary report. *Br. Med. J.*, **1**, 1451–1455.

DOLL, R. & HILL, A.B. (1956). Lung cancer and other causes of death in relation to smoking. A second report on the mortality of British doctors. *Br. Med. J.*, **2**, 1071–1081.

DOLL, R. & PETO, R. (1981). *The Causes of Cancer*. Oxford: Oxford University Press.

GILLIES, P.A. & GALT, M. (1990). Teenage smoking – fun or coping? In Wimbust, J.A.M. & Maes, S. (eds) *Lifestyles and Health: New Developments in Health Psychology*. DSWO/ LEIDEN. Netherlands: University Press.

GODDARD, E. (1990). *Why Children start Smoking*. London: HMSO.

HAMMOND, E.C. & HORN, D. (1958*a*). Smoking and death rates – report on forty-four months of follow-up of 187,783 men. I. Total mortality. *J. Amer. Med. Assoc.*, **166**, 1159–1172.

HAMMOND, E.C. & HORN, D. (1958*b*). Smoking and death rates – report of forty-four months of follow-up of 187,783 men. II. Death rates by cause. *J. Amer. Med. Assoc.*, **166**, 1294–1308.

JACOBSON, B. (1986). *Beating the Ladykillers. Women and Smoking*. London: Pluto Press.

LADER, D. & MATHESON, J. (1991). *Smoking among Secondary School Children in 1990*. London: Office of Population Censuses and Surveys, HMSO.

McNEIL, A.D., JARVIS, M.J., STAPLETON, J.A., RUSSELL, M.A.H., EISER, J.R., GAMMAGE, P. & GRAY, E.H. (1988). Prospective study of factors predicting uptake of smoking in adolescents. *J. Epidemiol. & Commun. Health*, **43**, 72–78.

MURRAY, M., SWAN, A.V., BEWLEY, B.R. & JOHNSON, M.R.D. (1984). The development of smoking during adolescence – the MRC/Derbyshire smoking study. *Internat. J. Epidemiol.*, **12**, 185–192.

MURRAY, M., KIRYLUK, S. & SWAN, A.V. (1985). Relation between parents' and children's smoking behaviour and attitudes. *J. Epidemiol. & Commun. Health*, **39**, 169–174.

NELSON, E. & CHARLTON, A. (1991). Does the voluntary agreement work? *Health Educat. J.*, **50**, 12–15.

OFFICE OF POPULATION CENSUSES AND SURVEYS (1991). General Household Surveys: *Cigarette Smoking, 1972–1990*. (SS91/3). London: O.P.C.S.

ROYAL COLLEGE OF PHYSICIANS (1962). *Smoking and Health*. London: Pitman Medical.

ROYAL COLLEGE OF PHYSICIANS (1977). *Smoking or Health*, Tunbridge Wells: Pitman Medical.

SWAN, A.V., MURRAY, M. & JARRETT, L. (1991). *Smoking Behaviour from Pre-Adolescence to Young Adulthood*. Aldershot: Avebury (Gower) Publishing Co.